# A Spatially Distributed Fiber-Optic Temperature Sensor for Applications in the Steel Industry

**DOI:** 10.3390/s20143900

**Published:** 2020-07-13

**Authors:** Muhammad Roman, Damilola Balogun, Yiyang Zhuang, Rex E. Gerald, Laura Bartlett, Ronald J. O’Malley, Jie Huang

**Affiliations:** 1 Department of Electrical and Computer Engineering, Missouri University of Science and Technology, Rolla, MO 65409, USA; mrhmc@mst.edu (M.R.); yz8r4@mst.edu (Y.Z.); geraldr@mst.edu (R.E.G.II); 2 Department of Material Science and Engineering, Missouri University of Science and Technology, Rolla, MO 65409, USA; dsbcrh@mst.edu (D.B.); lnmkvf@mst.edu (L.B.); omalleyr@mst.edu (R.J.O.)

**Keywords:** temperature sensor, Rayleigh scattering, optical frequency domain reflectometry, distributed sensing, optical fiber, peritectic behavior, metal casting, aluminum alloy

## Abstract

This paper presents a spatially distributed fiber-optic sensor system designed for demanding applications, like temperature measurements in the steel industry. The sensor system employed optical frequency domain reflectometry (OFDR) to interrogate Rayleigh backscattering signals in single-mode optical fibers. Temperature measurements employing the OFDR system were compared with conventional thermocouple measurements, accentuating the spatially distributed sensing capability of the fiber-optic system. Experiments were designed and conducted to test the spatial thermal mapping capability of the fiber-optic temperature measurement system. Experimental simulations provided evidence that the optical fiber system could resolve closely spaced temperature features, due to the high spatial resolution and fast measurement rates of the OFDR system. The ability of the fiber-optic system to perform temperature measurements in a metal casting was tested by monitoring aluminum solidification in a sand mold. The optical fiber, encased in a stainless steel tube, survived both mechanically and optically at temperatures exceeding 700 °C. The ability to distinguish between closely spaced temperature features that generate information-rich thermal maps opens up many applications in the steel industry.

## 1. Introduction

Steelmaking facilities require continuous temperature measurements throughout the manufacturing process, to ensure consistent product quality and high productivity. Real-time temperature monitoring in processes like furnace reheating, furnace annealing, continuous casting, rolling, and hot forming enables the use of advanced control strategies and provide process control feedback for the aforementioned processes. Temperature measurements in rolling mills help to control surface temperature and shape, thereby ensuring product quality. Temperature measurements in the tundish provide feedback to control temperature variations of the steel, to ensure a stable caster operation in the continuous casting of steel. A mold instrumented with a temperature measurement system deployed in a continuous casting process provides data that can be used to detect crack formations in the solidifying shell, avoid breakouts, and provides the opportunity to monitor and control heat transfer during solidification. 

In addition to the quality assurance of various metal products, temperature measurements are also a means of detecting process faults, to ensure the safety of equipment and workers. Temperature monitoring of material transfer conveyors can mitigate safety risks. Monitoring temperature in the refractory linings can help detect hot spots caused by refractory failures, to avoid catastrophic breakouts, thus enabling timely maintenance.

Monitoring temperature also leads to cost-effective production by providing more control over energy expenditures and providing the means for improving production efficiency. Processes like furnace annealing and reheating can benefit greatly from continuously monitoring temperatures at high spatial resolution.

Owing to the significance of continuous temperature measurements in steelmaking, an accurate, information-rich, fast, and minimally invasive temperature measurement system is highly desirable. A measurement system with such characteristics would be helpful in achieving safe and cost-effective steelmaking with good product quality and high productivity. Temperature measurements in steel industry applications are commonly performed with thermocouples [1,2,3,4]. Thermocouples provide reliable and fast temperature measurements with good accuracy and high-temperature resolution; however, they have several limitations. For instance, thermocouples provide single-point measurements, which make them less suited for applications that exhibit closely spaced temperature features. Therefore, the inability of thermocouples to easily perform distributed temperature measurements limits their utility in many important applications. Temperature mapping using multiple thermocouples makes the system cumbersome, due to the large number of lead wires needed to interrogate them. Moreover, the size of the thermocouple probe often requires a significant modification to the device being measured, possibly interfering with the desired measurement or compromising the structural integrity of the device. Thermocouples, due to their electrical conducting nature, are also affected by electromagnetic interference in applications that involve electromagnetic phenomena, such as magnetic stirrers, arc furnaces, and radiofrequency transmitters.

The aforementioned limitations of thermocouples motivated researchers to seek alternative sensing technologies. In this quest, fiber-optic sensing technologies were explored as potential solutions for temperature measurements. Optical fiber technologies have emerged as promising sensing solutions, due to the miniaturized size of the optical waveguide, immunity to electromagnetic interferences, and ability to withstand harsh environments. In addition, optical fibers afford the innate capability for distributed sensing [5]. Several optical fiber-based sensors for temperature measurements were reported. Fiber-optic interferometers are commonly exploited for temperature measurements [6]. Interferometers offer high sensitivity and good temperature resolution, but they can only provide point measurements, similar to thermocouples. Fiber-optic distributed temperature sensors based on optical time-domain reflectometry (OTDR) are widely studied [7,8,9,10]. These OTDR-based sensors offer a spatial resolution of the order of a few meters, which is not suitable for steel industry applications due to the closely spaced temperature features in the steelmaking processes. Fiber Bragg gratings (FBGs) are also used for distributed temperature sensing [11,12,13,14]. FBGs attracted considerable interest in the steelmaking industry, due to a quasi-distributed sensing capability with a reasonable spatial resolution (~1 cm), high-temperature sensitivity (~10 pm/°C), and a fast measurement rate (~5 kHz). Thomas and Okelman reported results using a casting mold with embedded FBG sensors for temperature and strain measurements [15]. The FBG sensors were embedded in a copper mold, using a nickel electroplating process. Temperature variations were recorded 1 mm away from the hotface, in a laboratory simulation of the continuous casting process. Lieftucht et al. developed a mold embedded with FBGs for temperature measurements in a continuous casting process of steel [16]. Temperature profiles were used to calculate mold levels and local heat flux readings. Spierings et al. designed a mold by embedding FBGs in the upper half of a copper mold plate with 2660 temperature measurement points [17]. The instrumented mold was tested on different steel grades. Temperature measurements were used to observe fluid flow and mold thermal behavior. Currently, there are several companies offering commercial systems for thermal monitoring of a mold, based on fiber Bragg gratings.

Although FBGs offer better spatial resolution than OTDR, they have other limitations. For example, FBGs are not truly distributed and they only afford quasi-distributed sensing. Moreover, FBGs require modifications to single-mode fibers to create the gratings, which add to the cost of the fiber. Another known disadvantage of FBGs is their inability to withstand temperatures exceeding 700 °C, as the gratings are erased at elevated temperatures. Optical frequency domain reflectometry (OFDR) based on Rayleigh scattering is another fiber-optic distributed sensing solution [18,19,20,21,22]. OFDR systems employ un-modified single-mode optical fibers as sensor devices. Moreover, OFDR systems offer spatial resolution in the range of submillimeter to a few millimeters, with fast measurement rates. The distributed sensing capability with a high spatial resolution and fast update rates (few hundred Hz) make OFDR an exciting prospect for the characterization of phenomena that involve closely spaced static and transient temperature features. Various fiber-optic distributed temperature sensors, based on the OFDR technology, were reported [23,24,25,26]. Yan et al. reported Rayleigh scattering-based fiber-optic sensors for real-time temperature monitoring of solid oxide fuel cells [24]. The spatially distributed temperature measurements were performed at up to 800 °C, with a 5-mm spatial resolution. Boyd et al. demonstrated a Rayleigh scattering-based fiber-optic sensor system to monitor distributed temperatures, along superconducting degaussing cables, in cryogenic environments [25]. Temperature measurements were performed along a 10 m long optical fiber, with a 7-mm spatial resolution.

In this work, spatially distributed fiber-optic temperature sensors based on Rayleigh scattering are proposed for applications in the steel industry. The temperature measurements can be performed along optical fibers, over distances of up to 50 m. The proposed sensor system can perform spatially distributed temperature measurements at up to 750 °C. Although the temperatures in some steelmaking processes can exceed 1600 °C, there are several applications in the steel industry where the maximum temperature is well below the maximum temperature measurement capability of the proposed system. One such example for a steel industry application is temperature measurements in a continuous caster mold. The studies reported in [15,16,17] showed that the temperatures inside the copper molds used in the continuous casting of steel are well below 500 °C. The maximum measurable temperature of the proposed sensor system is limited by the degradation of the Rayleigh backscattering signals at elevated temperatures. It was demonstrated in [27,28] that Rayleigh scattering-based sensing using the standard single-mode fiber could be used for temperature measurements of up to 750 °C. As the system temperature approached 750 °C, the Rayleigh backscattering signals degraded, possibly due to the increased mobility of the intrinsic defects in the optical fiber material. Another study demonstrated that the temperature measurement range of the Rayleigh scattering-based sensor system could be extended to 850 °C, using gold-coated single-mode fibers [29].

In this work, OFDR systems were employed to perform fiber-optic distributed temperature measurements. The fiber-optic temperature measurements were compared with conventional thermocouple measurements, validating the accuracy of the former and manifesting its distributed sensing capability. Moreover, experiments were designed and conducted to provide evidence that Rayleigh scattering-based fiber-optic temperature sensors, due to the high spatial resolution and the fast measurement rates of OFDR systems, are suitable candidates for thermal mapping of phenomena that involve closely spaced temperature features. The measurement capability and survivability of a fiber-optic temperature sensor in a metal casting was also tested. The temperature profile across the cavity of a sand mold was monitored during an aluminum pour and solidification. The experimental results showed that the distributed fiber-optic temperature sensor could be a potential candidate for temperature measurements in metal casting applications.

## 2. Sensing Principle and Interrogation System

### 2.1. Rayleigh-Scattering-Based Temperature Measurements

The proposed system of distributed temperature measurements is based on Rayleigh scattering in a single-mode optical fiber. Rayleigh scattering originates when light strikes an inhomogeneity in a continuum of matter, with dimensions smaller than the wavelength of light. In an optical fiber, random fluctuations in the refractive index of the optical fiber material (glass) cause Rayleigh scattering. Temperature variations cause changes in both the refractive index and the length of the optical fiber, which result in shifts in the Rayleigh backscattering (RBS) spectra. The RBS shift ∆*λ* caused by a temperature change ∆*T* can be expressed as:

(1) $$\frac{∆\lambda }{\lambda }=\left(\alpha +\xi \right)∆T$$

where *α* is the thermal expansion coefficient and *ξ* is the thermo-optic coefficient of the optical fiber material. The value of the thermal expansion coefficient is 0.55 × 10^–6^/°C, while that of the thermo-optic coefficient is 8.5 × 10^–6^/°C. As the value of the thermal expansion coefficient is approximately an order of magnitude smaller than the thermo-optic coefficient, the spectrum shift due to a temperature change is usually attributed to the change in the refractive index.

### 2.2. Optical Frequency Domain Reflectometry (OFDR)

**Figure 1:**
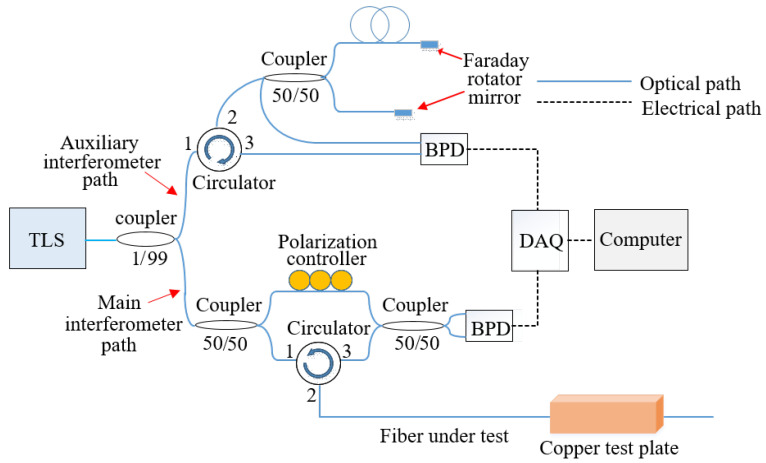
A schematic diagram of the optical frequency domain reflectometry system. TLS—tunable laser source, BPD—balanced photodetector, and DAQ—data acquisition card.

An OFDR interrogator system was used to acquire, process, and analyze backscattered light. In an OFDR system, light from a tunable laser source was launched into an optical fiber network containing an interferometer. The interference signal from the interferometer was detected and analyzed. As shown in the schematic of the OFDR system we developed in our laboratory (Figure 1), light from a tunable laser source (LUNA PHOENIX^TM^ 1200) with a 50 nm tuning range (1515 nm–1565 nm) and a 1000 nm/s tuning speed was launched into an optical fiber network. The first coupler split the incident light between the two arms—one beam going into an auxiliary interferometer and the other beam going into the main interferometer. The auxiliary interferometer was used as a clock generator for the data acquisition card, in order to compensate for the nonlinear sweep of the laser. The main interferometer was a Mach-Zehnder interferometer with two arms, one used as a reference arm and the other as a sensing arm. Backscattered light from the sensing arm combined with the reference signal in a 50–50 coupler and generated an interference signal, which was collected by a data acquisition card and then transmitted to a computer. Temperatures metered along the longitudinal spatial dimension of the optical fiber were retrieved through a series of signal processing steps.

## 3. Distributed Temperature Sensing Experiments

The purpose of this project was to develop a fiber-optic distributed temperature sensor system for applications in the steel industry. Preliminary tests were performed to demonstrate the distributed sensing capability of the fiber-optic temperature measurement system. In many such experiments, optical fibers were nested in metal tubes. Metal tubes were used as potential casings, anticipating the deployment of optical fibers in the steel industry processes. Moreover, to validate the accuracy of the Rayleigh-scattering-based fiber-optic temperature sensor, fiber-optic temperature measurements were compared with thermocouple measurements. As discussed earlier, the capability of distributed temperature measurement with high spatial resolution could be very useful for applications in the steel industry. The high spatial resolution of the sensing system makes it possible to measure localized temperature features. The ability to measure spatial variations in temperature adds great value for applications in the steel industry. Control experiments were performed to map closely spaced fluctuating temperature features. The closely spaced temperature variations were achieved by heating the test objects. The test objects were created by machining an air cavity into a copper plate. The copper plates were used as test objects due to their resemblance to copper molds, used in continuous steel casting. Results obtained from the thermal mapping experiments provided initial evidence that the fiber-optic system had the ability to distinguish between closely spaced temperature features. Furthermore, an aluminum solidification experiment was conducted to demonstrate the measurement capability and survivability of optical fibers in metal casting applications.

### 3.1. Optical Fiber Encased in a Copper Tube Equipped with Heating Coils

**Figure 2:**
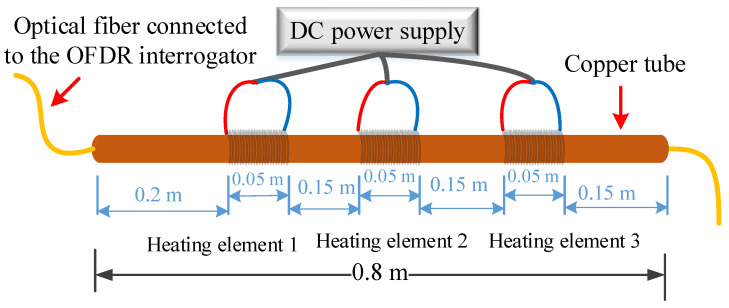
A schematic diagram of the optical frequency domain reflectometry (OFDR) system setup used for the distributed temperature measurements. The copper tube-encased optical fiber was connected to the OFDR interrogator. Three heating coils were used to create temperature gradients along the length of the copper tube.

A test experiment for distributed temperature measurements was performed by placing a single-mode optical fiber (o.d. = 250 µm) in a 0.8 m long copper tube (i.d. = 660 µm; o.d. = 1800 µm), as shown in Figure 2. The optical fiber was connected to the OFDR interrogator. Three heating elements, connected to a DC power supply, were used to locally heat three distinct regions of the copper tube. The heating elements used in the experiment were nichrome resistance wires. The heating elements were simultaneously powered by a DC power supply (Agilent E3616A), drawing approximately 1 A of current at a voltage of 15 V. When the power was turned on, the temperatures recorded by the fiber-optic system increased with an average ramp-up rate of 10–11 °C/min for the heating elements, before reaching a steady-state temperature profile. Temperature gradients were generated along the copper tube, due to the interplay between the heat generated by the heating elements and the radiation heat loss to the ambient environment. Distributed (longitudinal) temperature profiles along the optical fiber nested in the copper tube were recorded with a measurement period of 10 s, throughout the electric heating process.

**Figure 3:**
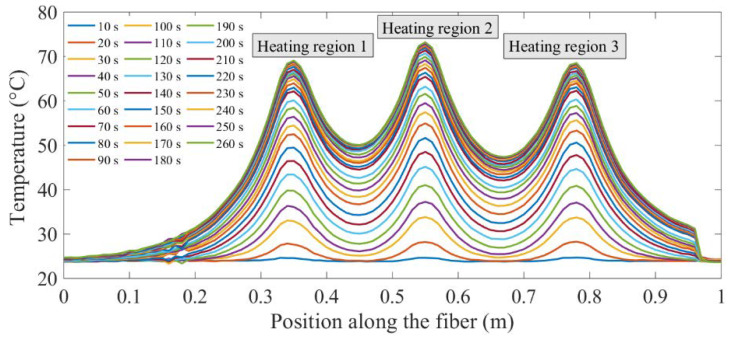
Spatial (longitudinal) temperature profile along the length of an optical fiber nested in a copper tube. Three peak temperature regions correspond to the location of the three heating coils placed along the length of the copper tube. A spatial temperature profile was recorded every 10 s during heating.

The temperature distribution along the optical fiber encased in the copper tube revealed three distinct regions of elevated temperatures in the center sections and lower temperatures at the end sections of the copper tube, as shown in Figure 3. At any one point along the copper tube, temperature measurements were made with a temperature measurement resolution of ±1 °C. Temperature measurements were recorded every 5 mm, along the length of the copper tube.

### 3.2. Comparison of Fiber-Optic Temperature Measurement System with Thermocouples

**Figure 4:**
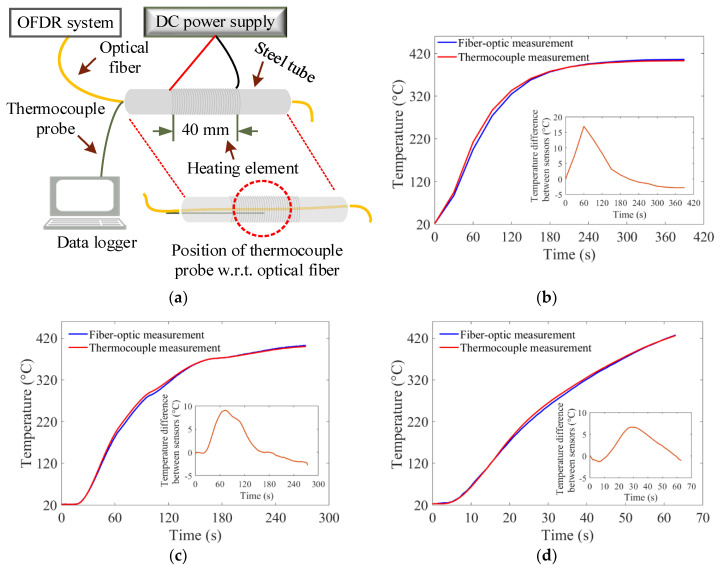
Test apparatus and single-point temperature measurement comparisons from fiber-optic and thermocouple measurements for a temperature-ramp experiment. (**a**) A schematic of the experimental setup used to compare fiber-optic with thermocouple measurements. The thermocouple junction was placed at the center of the heating coil. The corresponding position of the optical fiber was identified using localized point heating. (**b**) Temporal temperature profiles of fiber-optic and the thermocouple measurements. The setup was heated with an average ramp-up rate of 84 °C/min. The inset plot shows temperature differences between the two sensors over time. (**c**) Temporal temperature profiles of fiber-optic and thermocouple measurements. The setup was heated with an average ramp-up rate of 82 °C/min. (**d**) Temporal temperature profiles of fiber-optic and thermocouple measurements. The setup was heated with an average ramp-up rate of 356 °C/min.

After demonstrating the spatially-distributed sensing capability of the optical fiber sensor and interrogator system, experiments were carried out to compare fiber-optic temperature measurements with thermocouple measurements. In one of the experiments, an optical fiber (o.d. = 0.25 mm) was placed inside a 100-mm long steel tube (i.d. = 2 mm; o.d. = 3 mm), and a 40-mm long heating coil was used to heat the steel tube, as shown in Figure 4. A K-type thermocouple (probe diameter = 0.5 mm), connected to a data logger (Graphtec GL220), was placed inside the tube and positioned at the center location of the heating element, as shown in Figure 4a. The heating coil was powered by a DC power supply (Mastech HY3005D). Three temperature ramp-up experiments were performed to compare the temporal temperature profiles of fiber-optic and the thermocouple measurements. In the first experiment, when the power was turned on, the temperature increased with an average ramp-up rate of 84 °C/min, before reaching a steady-state temperature of 400 °C in 4.5 min. Temperature readings from both the fiber-optic and the thermocouple systems were recorded simultaneously at a measurement rate of one sample per second. Both the fiber-optic and the thermocouple systems performed temperature measurements with a resolution of less than ±1 °C. As discussed earlier, the maximum temperature measurement capability of the developed fiber-optic system was ~750 °C. The thermocouple system, on the other hand, could be used for temperature measurements of up to 1300 °C. Since the fiber-optic system provided distributed measurements along the length of the fiber, a single position on the optical fiber that was closest to the thermocouple was selected to compare the fiber-optic measurements with the thermocouple measurements. The position on the optical fiber, closest to the tip of the thermocouple probe, was identified using localized point heating, before the start of the experiment. The comparison of temperature measurements from both systems, over time, is shown in Figure 4b. The results demonstrated good agreement between the fiber-optic and thermocouple temperature measurements. The differences in temperature readings between the fiber-optic and the thermocouple systems at the early stages of the heating experiment, as depicted in Figure 4b, were due to the relative position of the thermocouple junction, with respect to the optical fiber inside the tube. However, both systems registered similar temperatures when the steady-state was reached, where a constant temperature was maintained inside the tube. The experiment was repeated an additional two times, with average ramp-up rates of 82 °C/min and 356 °C/min, respectively. The results from the experiments are shown in Figure 4c,d. The results demonstrated good agreement between the fiber-optic and thermocouple temperature measurements. In addition to the difference in the spatial positions of both sensors, temperature readings recorded with both systems could be different due to the different thermal responses of the optical fiber and the thermocouple probes. During the heating process, the fiber-optic system registered slightly lower temperatures than the thermocouple system, as evident from Figure 4b–d. The temperature differences between the two sensors, shown in the inset plots in Figure 4b–d, might be due to the fact that the thermal conductivity of the optical fiber was lower than that of the thermocouple. Therefore, the thermocouple responded more quickly to the changes in temperatures. More detailed work needs to be done to improve the apparatus used to compare fiber-optic with thermocouple measurements.

**Figure 5:**
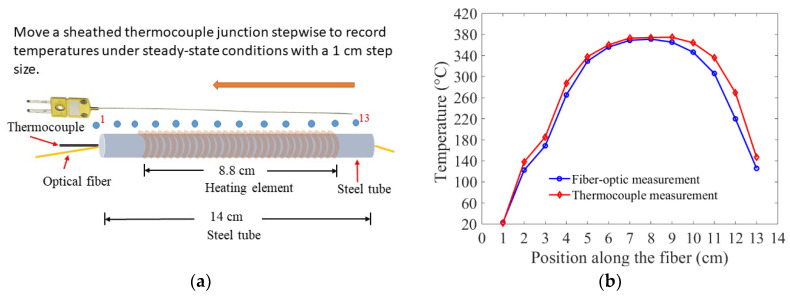
Comparison of a spatial temperature profile derived from fiber-optic measurements with corresponding thermocouple measurements. (**a**) Schematic of the setup used for spatial temperature mapping of fiber-optic measurements and thermocouple measurements. The optical fiber, connected to the OFDR interrogator, was nested in a steel tube. A K-type thermocouple was re-positioned, within the steel tube and adjacent to the optical fiber, from one end of the tube to the other, to record steady-state temperature readings at 13 different positions. (**b**) Superposition of the spatial temperature mapping of the fiber-optic measurements and thermocouple measurements. The fiber-optic temperature measurements at 13 different positions were compared with the corresponding thermocouple measurements and showed close agreements.

As discussed earlier, the inability of single-point thermocouples to perform distributed temperature measurements limits their application. This limitation was illustrated in an experiment where a spatial temperature profile obtained from a single continuous optical fiber nested in a metal tube was compared with a discrete spatial temperature profile that resulted from sequentially moving a thermocouple along the length of the same tube-encased fiber, as shown in Figure 5. In order to measure a spatial temperature profile using a conventional thermocouple, a sheathed K-type thermocouple was re-positioned from one end of the tube to the other in 1-cm steps, as illustrated in Figure 5a. The thermocouple readings, recorded under steady-state conditions, were compared with the corresponding fiber-optic temperature measurements. The two sets of temperature measurements were separated by small and variable temperature offsets, as shown in Figure 5b. Since the thermocouple probe was manually moved and re-positioned inside the tube, it was difficult to ensure that the corresponding position on the optical fiber was exactly the same as the location where the thermocouple junction (probe tip) was positioned. Therefore, the offsets that separated the two sets of temperature measurements were due to the variable relative positions of the thermocouple, with respect to the optical fiber. However, similar spatial temperature profiles were observed with both temperature measurement systems. This experiment reiterated the fact that measuring spatially-distributed temperatures with thermocouples require moving a single thermocouple stepwise to map out a series of spatially-disposed temperature measurements or; alternatively, using multiple thermocouples with a large number of signal wires would be required. In contrast, a single continuous fixed-position optical fiber can provide distributed temperature measurements along its length.

### 3.3. Thermal Mapping of Test Objects with Localized Temperature Variations

The ability to measure closely spaced temperature features is of great value for applications in the steel industry. Many steelmaking process configurations exhibit localized spatial and temporal variations in temperature. Information-rich temperature measurements would be very useful to characterize such phenomena. One such example for a steel industry application is spatial temperature measurements in a continuous caster mold. Inadequate mold lubrication, non-uniform shell growth, or crack formation in the shell can cause air gaps between a steel shell and the hotface of the mold, which leads to temperature fluctuations on the hotface of the mold [30]. Measuring these temperature fluctuations can be helpful in detecting and characterizing non-uniform shell growth events during casting. Several attempts were made to measure temperature variations on the exposed surface of the mold, caused by non-uniform shell growths using thermocouples and fiber-optic FBGs. However, the inability of thermocouple systems to measure closely spaced temperature features and the limited spatial resolution of the quasi-distributed FBGs are a big hurdle to map closely spaced temperature variations [2,16]. The present research demonstrates that Rayleigh-backscattering-based fiber-optic temperature sensors could be a potential solution to measure temperature fluctuations in the continuous casting mold.

#### 3.3.1. One-Dimensional (1D) Thermal Mapping

**Figure 6:**
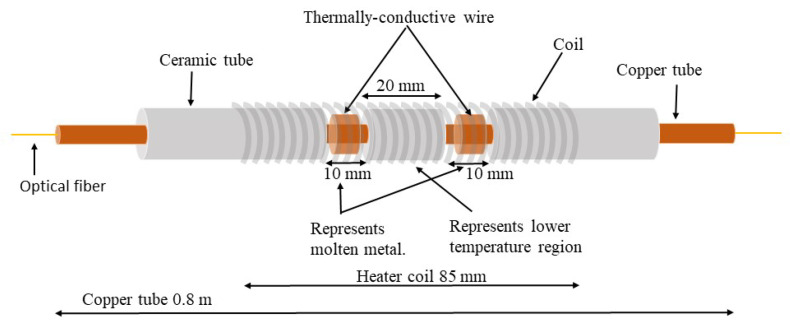
Copper tube assembly employed to create hot and cold spots along the long axis of an embedded optical fiber. An optical fiber was threaded through the copper tube. The ceramic tube in the middle was intended to create a low-temperature region by reducing heat flux from the heater coil to the optical fiber.

In an attempt to mimic the thermal behavior of a metal casting mold and demonstrate how well the fiber-optic system distinguishes closely spaced temperature features, hot and cold spots of known dimensions were created on a copper tube, as shown in Figure 6. An optical fiber, connected to the OFDR interrogator, was threaded through the copper tube. A ceramic tube located between two wire-wrapped copper regions was used to mimic an air gap similar to a gap caused by a non-uniform shell growth. The formation of an air gap at the metal–mold interface during casting caused reduced heat flux from the metal to the mold. Heat flux anomalies yield localized temperature features on the surface of the mold. Similarly, in the copper/ceramic tube arrangements, the ceramic tube encased regions should exhibit lower temperatures, due to reduced heat transfer from the heating coil to the fiber. To achieve localized variations in temperature along the length of a copper tube, the heat source (heater coil) needs to generate heat at a high rate. If the heat output rate of the coil is low, closely spaced temperature features can disappear due to the good thermal conductivity of the copper tube. In this experiment, an average temperature ramp-up rate of 15 °C/s was achieved and employed for conducting spatially-distributed temperature measurements. The copper tube assembly was heated using a heater coil connected to a DC power supply (Mastech HY3005D), drawing 2.6 A current at a voltage of 26 V (~65 W). The spatial temperature profile during heating was measured with the fiber-optic system.

**Figure 7:**
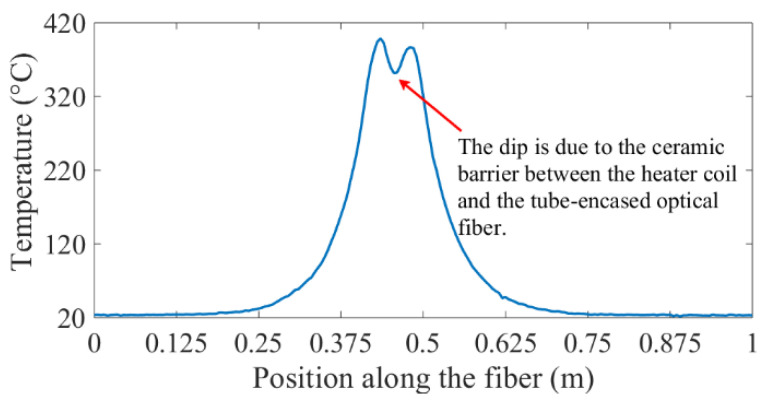
Spatially distributed temperature profile along the length of an optical fiber contained within a copper tube. The dip at the center of the temperature profile corresponded to a ceramic tube-encased region.

The measured spatially distributed temperature profile revealed a dip in temperature, as a result of the ceramic barrier between the heater coil and the copper tube, which contained the encased optical fiber sensor, as shown in Figure 7.

**Figure 8:**
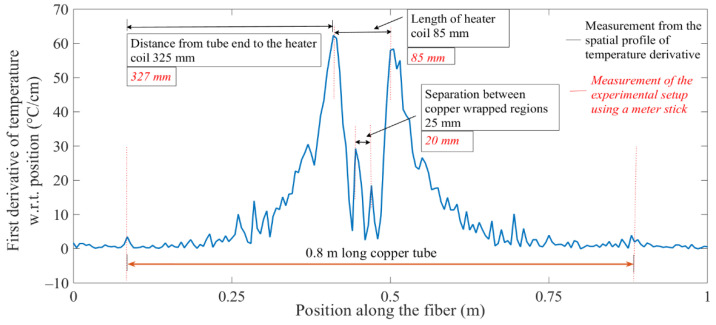
A plot of the absolute-value rectified spatial derivative of temperature versus position. Temperature features extracted from the plot are compared with the dimensions of the copper tube test setup with hot and cold spot features.

The plot in Figure 7 shows an obvious temperature feature, but quantifying the physical dimensions of the temperature feature from the plotted temperature data was not easy to carry out. The spatial derivative of the metered temperature versus position plot was calculated to address the limitations on facile interpretations of spatially distributed temperature profiles. Figure 8 shows the spatial derivative of temperature, along a sensor point position axis, which was configured with hot and cold spots, resulting in positive and negative peak features, due to sharp increases and decreases in temperature at the edges of the feature zones. The absolute value of the rectified spatial derivative yielded all positive peaks, which corresponded to the edges of the hot and cold spots. In this analysis, absolute-value rectified derivative plots were used to quantify the length of the lower temperature region, due to the easily identifiable sharp temperature changes at the ends of the lower temperature regions. Furthermore, the features extracted from the spatial derivative profiles of temperature were in good agreement with the dimensions of the test setup, as shown in Figure 8. This revelation demonstrated that the fiber-optic temperature sensor could distinguish between closely spaced hot and cold spots in a heated tube structure.

#### 3.3.2. Two-Dimensional (2D) Thermal Mapping

A series of experiments were conducted to demonstrate the 2D thermal mapping capability of the fiber-optic temperature measurement system. As discussed earlier, the capability of Rayleigh-backscattering-based fiber-optic temperature sensors to perform 2D thermal mapping could be useful for many applications in the steel industry. Real-time thermal mapping of the casting mold surface is an important process that can be used to determine the structural and mechanical properties of the solidifying shell. Information about various properties of the shell, such as surface roughness, cracks, shell buckling, and the resulting air gap formation at the metal–mold interface, could be obtained from the surface temperature of the mold [4,31]. Based on the temperature data of the mold, appropriate actions can be taken to improve the quality of the casting. There are many phenomena in steel casting that cause irregularities in shell growth. Peritectic behavior is one such phenomenon that leads to surface defects and breakouts in continuous casting. Peritectic behavior occurs during solidification of certain alloy systems, in which a secondary phase grows on the periphery of a primary phase. During the solidification of steel, for example, the primary phase–ferrite (δ) precipitates first from the molten metal. This pre-existing ferrite phase then reacts with the residual liquid metal to form the secondary solid phase–austenite (γ). The two solid phases formed during solidification have different thermal contractions, due to their different packing densities, which makes peritectic steel grades susceptible to shrinkage during solidification. The shrinkage caused by the δ/γ phase transformation during the initial solidification of peritectic steel grades can induce shell buckling, which in turn causes uneven shell formation, non-uniform shell and mold temperatures, and an average decrease in heat flux from shell to the mold [30]. Measuring closely spaced temperature features on the mold surface can be useful in the characterization of peritectic behavior. 

**Figure 9:**
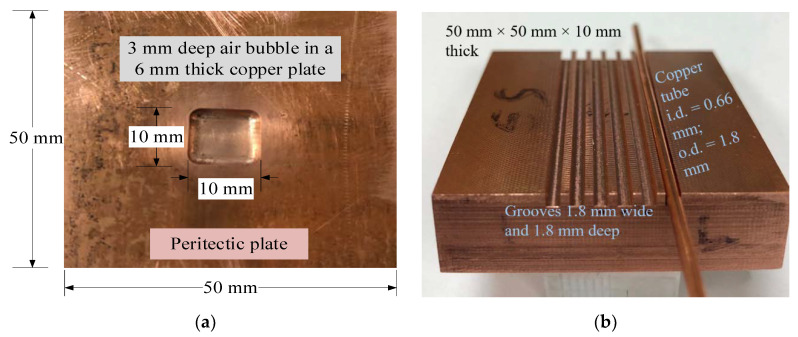
The arrangement of a peritectic plate model and a copper block with embedded optical fibers. (**a**) A peritectic plate model with an air bubble. The peritectic plate exhibits a lower temperature in the middle due to the presence of an air bubble. (**b**) A copper block equipped with fiber-optic temperature sensors. The grooves are machined on the surface of the block. The copper tube-encased optical fibers were press-fitted in the grooves.

In order to experimentally simulate the peritectic behavior in a lab environment, temperature mapping experiments were conducted using a *peritectic plate* model and a copper block equipped with optical fibers, as shown in Figure 9. The peritectic plate model (50 mm × 50 mm × 6 mm thick) with localized temperature variations was machined to mimic the behavior of a shell with non-uniform features. The copper plate of uniform thickness was modified by milling out a cubical bubble to simulate an air gap caused by non-uniform shell growth, as shown in Figure 9a. Another copper block was equipped with optical fibers (OF). Optical fibers were placed in copper tubes (i.d. = 0.66 mm; o.d. = 1.8 mm), which in turn were fitted in grooves (1.8 mm wide and 1.8 mm deep) machined on the surface of the copper block, as shown in Figure 9b.

**Figure 10:**
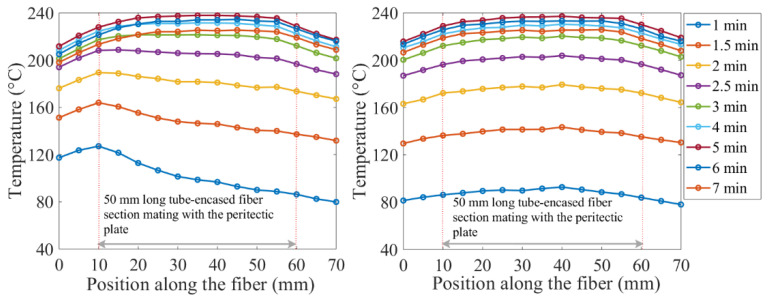
Temperature profiles from two sections of the optical fiber metered by mating the OF-equipped block with a peritectic plate model. Temperature features were subdued because the optical fiber was free-floating inside the copper tube. The copper tube was in contact with the hotface of the copper block.

The OF-equipped block had copper-tube-encased optical fibers in two grooves. In one of the experiments, the pre-heated peritectic plate with localized temperature variations was mated with the copper block equipped with optical fibers. When the block mated with the pre-heated peritectic plate, one of the sensor-equipped grooves was placed on the continuous flat metal surface of the peritectic plate, while the other groove had a section laid over the air bubble. The temperature distribution over the OF-equipped copper block, as a result of heat transfer from the peritectic plate to the OF equipped block, was measured. The section of the optical fiber laid over the air bubble was expected to show a dip in the spatial temperature profile. As shown in Figure 10, the temperature features were not prominent enough to reveal the physical dimensions of the test setup. The free-floating fiber inside the copper tube was suspected to be the cause of ambiguities in temperature measurements, due to the poor thermal contact between the optical fiber and the copper tube that was in contact with the hotface of copper block.

**Figure 11:**
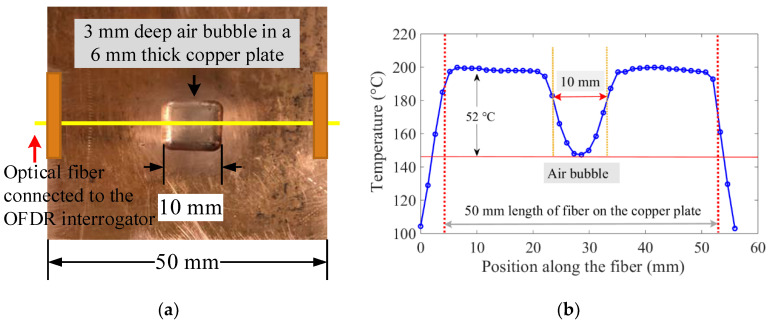
1D thermal modeling experiment using the peritectic plate model. (**a**) A section of an optical fiber taped on the surface of the peritectic plate. The dimensions of the air bubble were 10 mm × 10 mm × 3 mm deep. A 10 mm-long bubble in the center of the 50-mm long plate creates a low-temperature region. (**b**) Distributed temperature profile with a temperature dip in the middle. The 10 mm section of the optical fiber in the middle of the cavity does not make physical contact with the peritectic plate and registers lower temperatures.

To test the hypothesis that temperature features were subdued due to poor thermal contact between the free-floating optical fibers and the hotface of the copper block, another 1D thermal simulation experiment was performed using the same peritectic plate model into which a cubical bubble was machined, as shown in Figure 11. A bare optical fiber was taped onto the peritectic plate bridging the air bubble, as shown in Figure 11a. The peritectic plate was then heated on a hot plate. Temperature measurements were conducted with a 1.3 mm spatial resolution. The temperature profile measured in this experiment, as shown in Figure 11b, exhibited a low-temperature feature that corresponded well to the length dimension of the rectangular air bubble on the model sheet. This experiment demonstrated that the high thermal conductivity of a copper tube used in the earlier experiment most likely eradicated temperature gradients, making it difficult for the optical fiber to distinguish closely spaced temperature features. On the other hand, the bare fiber worked very well because the optical fibers were fabricated from glass, a thermally insulating material.

**Figure 12:**
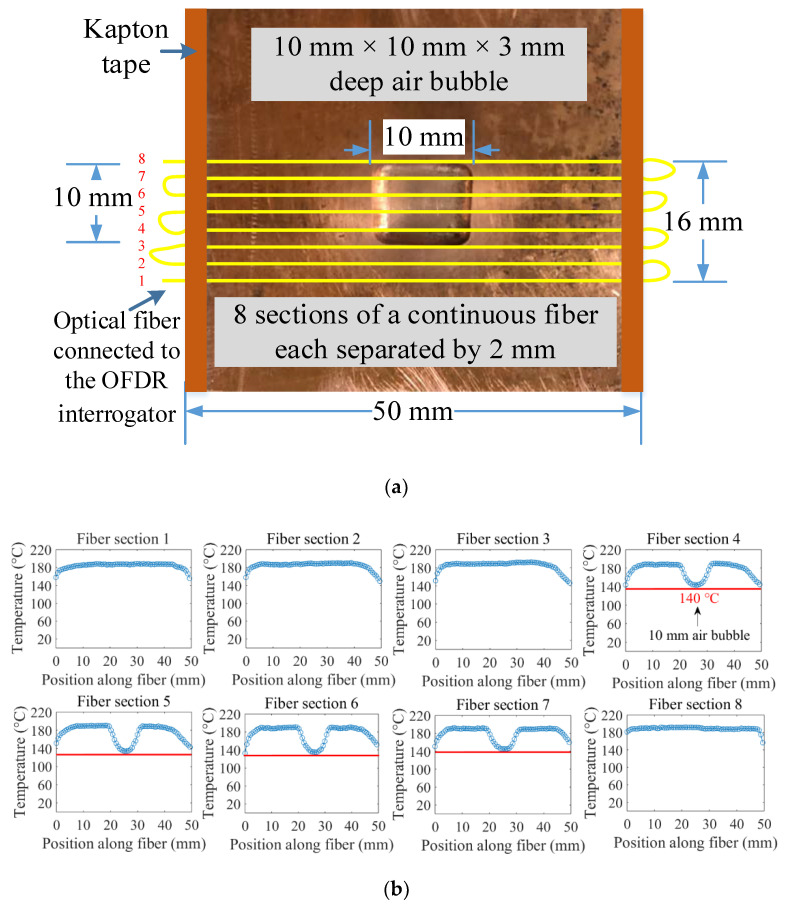
An arrangement of eight sections of an optical fiber on the peritectic plate and spatial temperature profiles along the sections of the fiber. (**a**) Arrangement of eight sections of a looped continuous optical fiber on the surface of the peritectic plate model. Four sections (1–3, 8) of the optical fiber were laid on the flat surface, while the other four sections (4–7) were laid over the air bubble. (**b**) Temperature distributions along eight 50 mm-sections of the optical fiber. The sections of the optical fiber laid over the air bubble exhibited a dip in the spatial temperature profile.

Another test was carried out to mimic the 2D thermal behavior of the mold surface with non-uniform shell growth. A 2D array, constructed using a single fiber-optic sensor, was attached to the peritectic plate model. The bottom side of the optical fiber-equipped peritectic plate arrangement was heated on a hot plate, and spatial temperature profiles along the fiber sections were recorded with the OFDR system, as shown in Figure 12. A continuous optical fiber was used to arrange eight parallel paths, each separated by 2 mm, on the peritectic plate model, as shown in Figure 12a. The peritectic plate was heated on a hot plate, and spatially distributed temperature data sets were recorded at constant time intervals. The temperature distribution along the sections of the optical fiber positioned on the flat surface registered a uniform temperature, while the sections of the optical fiber positioned along the top of the air bubble exhibited dips in the temperature profiles, as depicted in Figure 12b.

**Figure 13:**
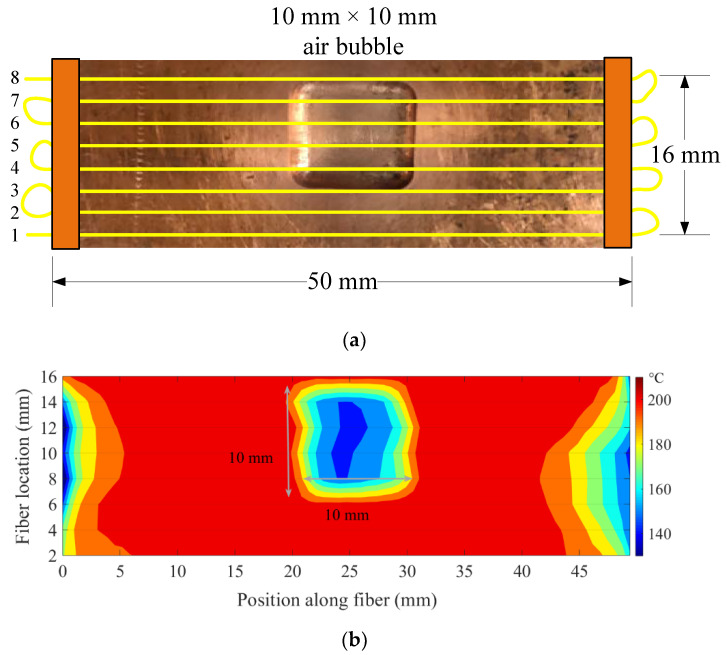
A cropped view of the peritectic plate overlaid with fiber-optic temperature sensors and a thermal map of the plate. (**a**) Arrangement of optical fibers on the peritectic plate with an air bubble in the center (10 mm × 10 mm). (**b**) Thermal map of the peritectic plate. The low-temperature feature is due to the air bubble.

Figure 13 illustrates a cropped view of the peritectic plate overlaid with fiber-optic temperature sensors and a thermal map of the plate recorded with the OFDR system. The thermal map of the peritectic plate matched well with the dimensions of the plate. The air bubble on the peritectic plate shown in Figure 13a appeared as a low-temperature feature on the thermal map of the plate. The dimensions of the low-temperature region shown in Figure 13b corresponded to the physical dimensions of the air bubble on the peritectic plate.

**Figure 14:**
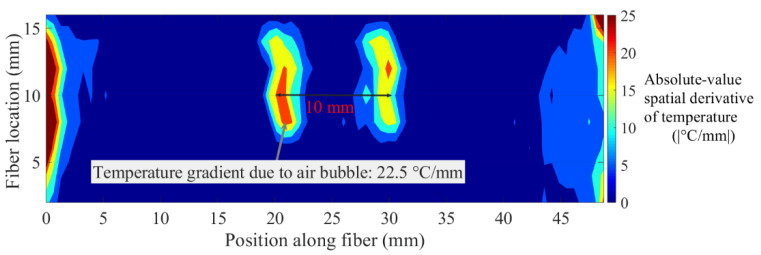
Contour plot of the absolute-value rectified spatial derivative of 2D temperatures. The two contour lines in the middle of the plot were caused by peaks in the absolute-value derivative plot. The separation between the two line features was the actual length of the low-temperature region.

As discussed earlier, absolute-value rectified spatial derivatives of temperature are useful to quantify the dimensions of hot and cold spots. Similarly, a contour plot of absolute-value rectified spatial derivative of temperature with respect to the position along the optical fiber could also be used to quantify the dimensions of the temperature features. In the aforementioned test, absolute-value rectified spatial derivatives of temperature, with respect to fiber position, resulted in peaks at the edges of the air bubble, due to sharp changes in temperature at those positions. The contour of absolute-value rectified spatial derivatives should give lines, one at each edge. The distance between these lines should be the length of the low-temperature region. The contour of the absolute-value rectified spatial derivative of temperature is given in Figure 14. The peak features in the contour plot appeared as two lines, which were in register with the edges of the low-temperature region. The width of each line was caused by the width of the derivative peak.

**Figure 15:**
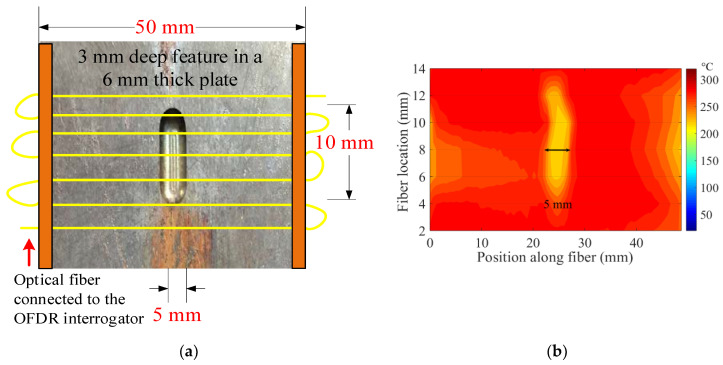
A top view of the peritectic plate overlaid with fiber-optic temperature sensors and a thermal map of the plate recorded with the OFDR interrogator. (**a**) Arrangement of a continuous optical fiber on a peritectic plate with a 5 mm-wide cylindrical air bubble. Seven sections of a continuous optical fiber were laid on the plate side-by-side, with 2-mm spacing between the adjacent sections of the optical fiber. (**b**) The thermal map of the peritectic plate model showed a low-temperature feature, due to an air cavity on the plate. The temperature feature matched well with the actual dimensions of the air cavity.

To demonstrate the sensitivity of the Rayleigh-backscattering-based temperature measurement, another peritectic plate model was machined by milling a bubble smaller than the one presented in the previous experiment (see Figure 11). A cylindrical air bubble, 3 mm in depth, 5 mm in width, and 10 mm in length, was milled out from the surface of a copper plate. Figure 15 illustrates a top view of the peritectic plate, overlaid with fiber-optic temperature sensors and a thermal map of the plate, recorded with the OFDR interrogator. A 2D sensor array, composed of seven sections of a continuous optical fiber, was laid on the peritectic plate model, as shown in Figure 15a. The bottom side of the optical fiber-equipped peritectic plate arrangement was heated on a hot plate, and a 2D thermal map of the plate was recorded. The low-temperature feature, shown in Figure 15b, matched well with the actual dimensions of the air bubble on the peritectic plate.

The experimental simulation of the peritectic-like phenomenon provided strong evidence that the Rayleigh-scattering-based fiber-optic temperature sensors could measure temperature features in a mold associated with peritectic behavior. The spatially continuous thermal maps of the mold during solidification could help monitor rapid and closely spaced thermal events at the meniscus and over the entire mold surface, which were attributed to the peritectic reaction.

### 3.4. Distributed Temperature Measurements across a Mold Cavity during Aluminum Casting

**Figure 16:**
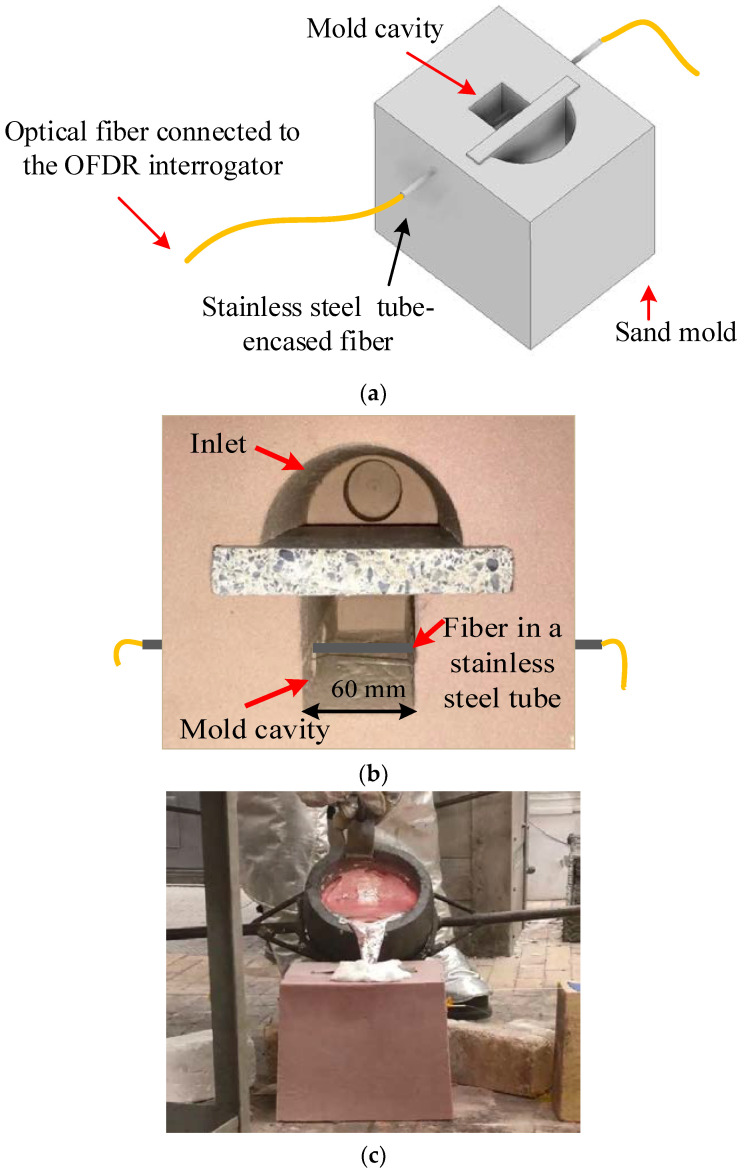
A schematic and photographs of a test setup that employed the Rayleigh-scattering-based fiber-optic sensor to investigate spatial and temporal temperature features in the process of an aluminum casting. (**a**) A schematic diagram of the experimental setup used for monitoring temperature across the mold cavity at a location approximately in the middle of the mold, during the process of pouring aluminum and subsequent solidification. (**b**) A top-view photograph of the sand mold used in the experiment, illustrating the location of the transverse fiber-optic temperature sensor. Molten metal was poured into the sand mold through an inlet, and the mold cavity was filled from the bottom using a baffle. The optical fiber, encased in a stainless steel tube, was placed laterally across the 60-mm wide mold cavity approximately in the mid-position in the mold cavity. (**c**) A photograph of the molten aluminum being poured into the mold.

The temperature measurement capabilities of the Rayleigh-scattering-based fiber-optic sensor were demonstrated in an aluminum casting experiment. Figure 16 illustrates a schematic and photographs of a test setup that employed the Rayleigh-scattering-based fiber-optic sensor to investigate the spatial and temporal temperature features in the process of aluminum casting. Temperature measurements were performed in a sand mold, during mold filling and solidification, using the apparatus illustrated in Figure 16a. An optical fiber temperature sensor was encased in a stainless steel tube (i.d. = 0.5 mm, o.d. = 0.8 mm). The rigid stainless steel tube protected the optical fiber and prevented it from kinking during the mold filling process. The tube-encased fiber was placed laterally in a 60-mm wide mold cavity, across the melt-flow direction, as illustrated in Figure 16b. An aluminum alloy (Al 90.062%, Si 7.350%, Cu 1.346%, Fe 0.457%, Mg 0.341%, Zn 0.289%, Mn 0.056%, and Ti 0.010%) was induction melted, heated to 730 °C, and hand ladled, to bottom-fill the instrumented sand mold, as shown in Figure 16c.

**Figure 17:**
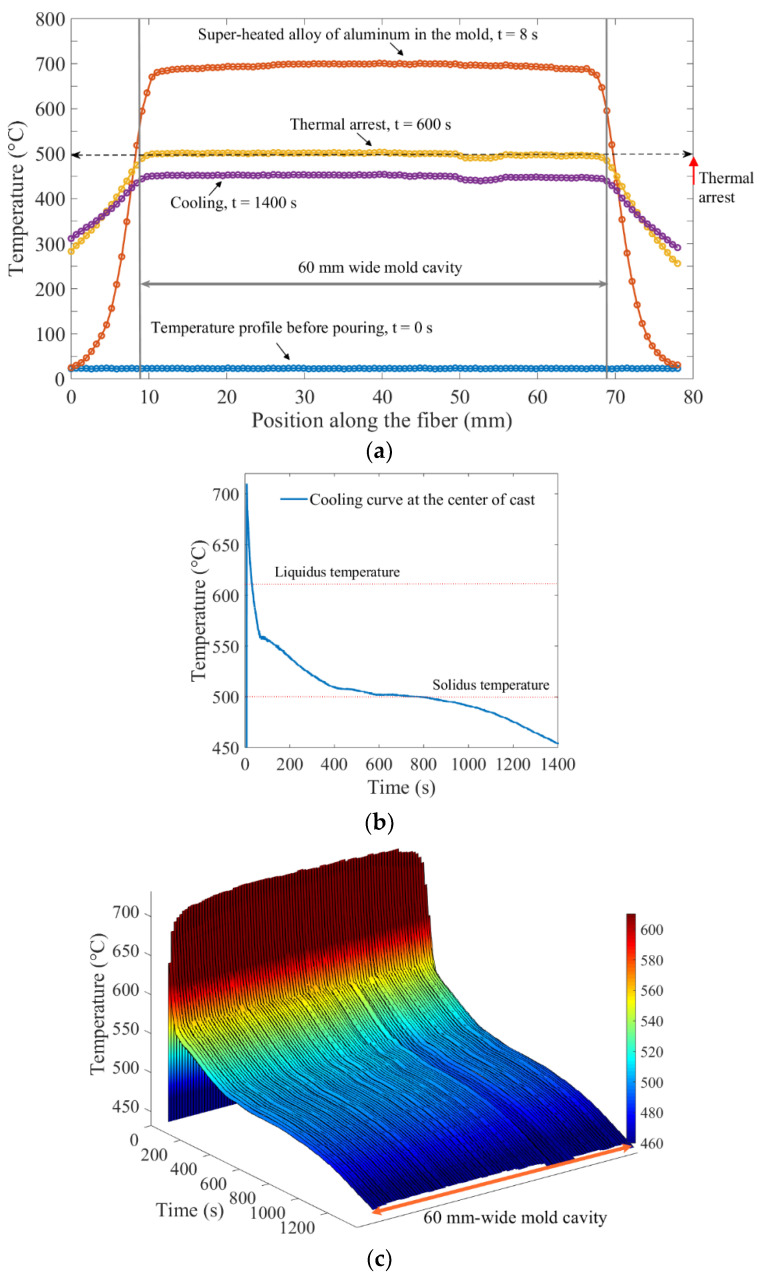
Temperature measurements across the mold cavity recorded with the OFDR interrogator, during the process of pouring aluminum and the subsequent solidification. (**a**) Spatially-distributed temperature profile across the mold cavity. The time *t* = 0 s marked the moment just before the molten metal was poured into the mold cavity. At time *t* = 8 s, the mold was completely filled with the molten aluminum. At time *t* = 600 s, the thermal arrest temperature profile was observed at 500 °C. The time *t* = 1400 s corresponded to the last recorded measurement during the cooling process. (**b**) The cooling curve recorded at the center of the cast. Different temperatures, such as liquidus and solidus, could be observed from the cooling curve. (**c**) Spatial-temporal thermal map showing cooling curves at 92 equally spaced positions, across the 60 mm-wide mold cavity.

Figure 17 illustrates temperature measurements across the mold cavity during mold filling and solidification. The spatially distributed temperature profiles along the width of the mold cavity and through the mold wall, at different times, are shown in Figure 17a. The metered temperatures along the tube-encased optical fiber jumped to approximately 710 °C, when the tube came in direct contact with the molten aluminum. The spatial temperature profile then dropped to about 500 °C where it exhibited a thermal arrest. The cool-down process was monitored down to 450 °C.

Figure 17b shows a cooling curve obtained at a location approximately at the center of the cast. The solidification started at the liquidus temperature (~610 °C) with the formation of primary (α-Al) dendrites, followed by eutectic reactions. The solidification ended at the solidus temperature (~500 °C). The multiple stages of solidification between liquidus (~610 °C) and solidus (~500 °C) could be distinguished by different gradients, during the phase transformations. After the completion of solidification, cooling resumed at a steady rate. The steady cooling rate after the solidification process indicated the uniform cooling of the solid.

As demonstrated earlier, Rayleigh-scattering-based fiber-optic sensors offer spatially continuous temperature measurements. The capability of fiber-optic sensors to perform distributed measurements cannot be easily replicated by conventional thermocouples. The obvious advantages of fiber-optic measurement are illustrated in Figure 17c, where the cooling curves across the entire mold cavity dimension are shown. Given the 0.65 mm spatial resolution of the fiber-optic measurement system, a single section of optical fiber 60 mm in length provided temperature profile measurements equivalent to temperature measurements from 92 thermocouples along the 60 mm-wide mold cavity. Such information-rich measurements can be helpful in investigating the internal temperature state of melts, during the solidification process. The heating and cooling curves at different locations within the mold could be obtained. The interfacial thermal resistance at the metal–mold interface could also be quantified using spatially distributed temperature profiles.

## 4. Conclusions

A Rayleigh-backscattering-based fiber-optic temperature sensing system employing an optical frequency domain reflectometry (OFDR) interrogator was developed. The distributed sensing capability of the system was tested in a foundry laboratory. The robust sensor head, composed of an unmodified single-mode fiber, survived temperatures up to 710 °C. The fiber-optic sensor produced accurate temperature measurements comparable to a standard thermocouple. Moreover, the spatially distributed sensing capability of an optical fiber enabled mapping of localized variations in temperatures. Spatially distributed fiber-optic temperature measurements provided a clear advantage over conventional thermocouples, due to the minimally invasive and information-rich distributed sensing capabilities of optical waveguides. Experiments were designed to provide evidence that the fiber-optic sensor was a viable candidate for temperature measurements in steel industry applications. Spatially-localized temperature variations were created on copper tube and plate test objects. Spatially localized temperature variations were successfully measured with the fiber-optic distributed temperature sensor with a 0.65-mm resolution. Temperature features were used to specify the dimensions of hot and cold spots. High spatial resolution and fast measurement rates (~250 measurements per second) by the fiber-optic system were useful in determining thermal maps of the copper test objects. Moreover, experiments revealed that optical fibers require continuous thermal contact with the copper test object, for reliable temperature measurements. In addition to experimental simulations using copper test objects, a metal casting experiment was conducted to test the measurement capability and survivability of optical fibers in harsh environments. The temperature profile across the cavity of a sand mold was successfully monitored, during an aluminum pour and solidification process. The aluminum solidification experiment provided a glimpse into how useful distributed fiber-optic temperature measurements could be for metal casting. Given the aforementioned advantages of distributed sensing with a high spatial resolution and a fast measurement rate, Rayleigh-backscattering-based fiber-optic sensors offer practical advantages for temperature measurement profiles in steel industry applications.
